# Comparative analysis of gene transcripts for cell signaling receptors in bone marrow-derived hematopoietic stem/progenitor cell and mesenchymal stromal cell populations

**DOI:** 10.1186/scrt323

**Published:** 2013-09-16

**Authors:** Khairul Anam, Thomas A Davis

**Affiliations:** 1 Regenerative Medicine Department, Operational and Undersea Medicine Directorate, Naval Medical Research Center, 503 Robert Grant Avenue, Silver Spring, MD 20910, USA

## Abstract

**Introduction:**

Knowing the repertoire of cell signaling receptors would provide pivotal insight into the developmental and regenerative capabilities of bone marrow cell (BMC)-derived hematopoietic stem/progenitor cells (HSPCs) and bone marrow mesenchymal stromal cells (BMMSCs).

**Methods:**

Murine HSPCs were enriched from fluorescence-activated cell sorting (FACS)-sorted Lin^–^c-Kit^+^Sca-1^+^ BMCs isolated from the tibia and femoral marrow compartments. Purified BMMSCs (CD73^+^, CD90^+^, CD105^+^, and CD45^–^, CD34^–^, CD31^–^, c-Kit^–^) with extensive self-renewal potential and multilineage differentiation capacity (into different mesodermal cell lineages including osteocytes, chrondrocytes, adipocytes) were derived from adherent BMC cultures after CD45^+^ cell depletion. Adherent colony-forming cells were passaged two to three times and FACS analysis was used to assess cell purity and validate cell-specific surface marker phenotype prior to experimentation. Gene transcripts for a number of cell signaling molecules were assessed using a custom quantitative real-time RT-PCR low-density microarray (94 genes; TaqMan^®^ technology).

**Results:**

We identified 16 mRNA transcripts that were specifically expressed in BMC-derived HSPC (including Ptprc, c-Kit, Csf3r, Csf2rb2, Ccr4, Cxcr3 and Tie-1), and 14 transcripts specifically expressed in BMMSCs (including Pdgfra, Ddr2, Ngfr, Mst1r, Fgfr2, Epha3, and Ephb3). We also identified 27 transcripts that were specifically upregulated (≥2-fold expression) in BMMSCs relative to HSPCs (Axl, Bmpr1a, Met, Pdgfrb, Fgfr1, Mertk, Cmkor1, Egfr, Epha7, and Ephb4), and 19 transcripts that were specifically upregulated in HSPCs relative to BMMSCs (Ccr1, Csf1r, Csf2ra, Epor, IL6ra, and IL7r). Eleven transcripts were equally expressed (<2-fold upregulation) in HSPCs and BMMSCs (Flt1, Insr, Kdr, Jak1, Agtrl1, Ccr3, Ednrb, Il3ra, Hoxb4, Tnfrsf1a, and Abcb1b), whilst another seven transcripts (Epha6, Epha8, Musk, Ntrk2, Ros1, Srms, and Tnk1) were not expressed in either cell population.

**Conclusions:**

We demonstrate that besides their unique immunophenotype and functional differences, BMC-derived HSPCs and BMMSCs have different molecular receptor signaling transcript profiles linked to cell survival, growth, cell differentiation status, growth factor/cytokine production and genes involved in cell migration/trafficking/adhesion that may be critical to maintain their pluripotency, plasticity, and stem cell function.

## Introduction

Adult stem cells are rare cell populations within specific tissues defined by their ability to undergo both self-renewal and differentiation. These tissue-specific stem cells are responsible for maintaining, generating, and replacing terminally differentiated cells of their host tissue as a consequence of physiologic cell turnover and or tissue damage due to injury [1,2]. Hematopoietic stem/progenitor cells (HSPCs) are functionally defined by their ability to self-renew and to contribute to all mature blood cell lineages [3]. Interestingly, HSPCs may contribute to nonhematopoietic tissues including the muscle, heart, brain and gut [4-7], which suggests an immense plasticity of differentiation and has raised the possibility of their use in tissue repair–regeneration [2]. Additionally, bone marrow and virtually all postnatal tissues contain small numbers of self-renewal multipotent adherent stromal–mesenchymal stem cells (MSCs) that have the potential to give rise to cells of diverse cell lineages, play a pivotal role in tissue repair–regeneration and have demonstrated nonimmunogenicity and potent immunomodulatory effects [8-10]. Furthermore, bone marrow-derived MSC (BMMSCs) have been shown to facilitate the *in vivo* engraftment of HSPCs and expansion of HSPCs in co-culture systems when used as feeder cells [11,12].

The self-renewal and differentiation of stem cells is probably subject to external modulation through receptors for a wide range of mediators including growth factors, cytokines, and chemokines. Furthermore, the potential diverse developmental plasticity of both HSPCs and BMMSCs to repair–replace damaged tissue suggests that local environmental factors and extrinsic influences drive stem cell differentiation and determine the function fate of these cells. Identification of the factors at the cellular and molecular levels that regulate the survival, proliferation, and development of these cells remains of key importance in identifying and propagating clinically relevant cell populations with diverse pathways of differentiation and therapeutic immunoregulatory potential.

Protein tyrosine kinase (PTK) networks are essential components of cell signaling pathways and play critical roles in cell proliferation, growth, development, metabolism and anti-apoptotic signaling, wherein they function to detect, amplify, filter and process environmental as well as intercellular signals [13]. PTKs include both transmembrane receptor tyrosine kinases (RTKs) and soluble cytoplasmic enzymes known as non-RTKs. In humans, 90 PTKs have been identified to date, comprising 58 RTKs and 32 non-RTKs [14]. Expression of most PTKs may be tightly regulated to retain unique features of a specific cell type. Characterizing the repertoire of high-affinity cell surface receptors for many growth factors, cytokines, chemokines and hormones might lead to be better understanding of the molecular phenotype and cell signaling pathways underlying the functional distinctions of bone marrow-derived HSPC and BMMSC populations.

The transcriptome of adult HSPCs and stromal stem/progenitor cells has been previously studied by other groups using high-density cDNA microarray hybridization techniques to comparatively decipher genes in undifferentiated cells and in developmentally regulated cell types involving various cellular processes including cell cycle, cell differentiation and cell proliferation [15-18]. Moreover, Son and colleagues investigated the expression profiles of PTK genes in undifferentiated and differentiated human embryonic stem cells [19]. High-density microarrays are an excellent tool for initial target discovery, but not the best tool for evaluating differential gene expression, whereas RT-PCR is often referred to as the gold standard for gene expression measurements [20,21]. In this study, we compared the gene expression profile of mRNA transcripts associated with signal transduction in bone marrow-derived undifferentiated highly purified Lin^–^ckit^+^Sca-1^+^ cells (LKSs) with BMMSCs using quantitative real-time RT-PCR (qRT-PCR), TaqMan^®^ low-density array analysis (96 genes of interest including controls). Both sets of cells significantly differed in expression of key transcripts for RTKs, non-RTKs, cytokine-growth receptors, G-protein coupled receptors, and several other cell signaling molecules.

## Methods

### Animals

Five-week-old to six-week-old BALB/c mice were purchased from the National Cancer Institute (Fredrick, MD, USA) and housed in pathogen-free animal facilities at the Walter Reed Army Institute of Research (Silver Spring, MD, USA), which is accredited by the Association for the Assessment and Accreditation of Laboratory Animal Care International. All procedures were conducted using facilities and protocol approved by the Animal Care and Use Committee of Walter Reed Army Institute of Research (protocol #K07-05). Mice were housed five animals per cage prior to use. Mice were used for experimentation at 8 to 12 weeks of age. Animal rooms were maintained at 21 ± 2°C with 50 ± 10% humidity on a 12-hour light/dark cycle. Commercial rodent ration (Harlan Teklad Rodent Diet 8604;) was available freely, as was acidified (pH 2.5) water to prevent opportunistic infections.

### Isolation of hematopoietic stem/progenitor cells

Purified HSPCs were obtained by the modification of the method described by Davis and colleagues [22]. Briefly, three mice were killed and the femurs and tibias were aseptically removed per experiment (*n* = 6 separate experiments). Bone marrow cells (BMCs) were flushed from the shaft with wash buffer consisting of Dulbecco’s phosphate-buffered saline supplemented with 2% heat-inactivated fetal calf serum (Hyclone, Logan, UT, USA), and penicillin (100 U/ml) and streptomycin (100 μg/ml) (culture reagents from Invitrogen, Rockville, MD, USA). BMCs were filtered through a nylon-mesh 70 μm cell strainer filter (BD Biosciences, San Diego, CA, USA) to produce a single cell suspension. After washing, BMCs were treated with ACK lysing buffer (NH_4_Cl; Invitrogen) and then incubated in a lineage antibody cocktail of biotin-conjugated anti-mouse mAbs specific for CD4, CD8, CD45RA/B220, CD11b, Gr-1 and Ter-119 (Miltenyi Biotec, Auburn, CA, USA) for 15 minutes at 4 to 12°C. After wash and cell resuspension steps, labeled Lin^+^ cells were incubated with anti-biotin magnetic microbeads and depleted by magnetic cell sorting (Miltenyi Biotec). Collected lineage-negative cells (Lin^–^) were then stained either with rat anti-mouse phycoerythrin (PE)-conjugated CD117 (c-Kit), APC-Cy7-conjugated CD45, fluorescein isothiocyanate (FITC)-conjugated Ly-6A/E (Sca-1) antibodies and PerCP-conjugated streptavidin to detect residual Lin^+^ cells or with control isotype-matched irrelevant mAbs labeled with the corresponding fluorochromes (BD-Pharmingen, San Diego, CA, USA). Cell sorting for LKSs was performed using a BD fluorescence-activated cell sorting (FACS) Aria II flow cytometer (Becton Dickinson, San Jose, CA, USA). Reflow analysis of sorted cells to check purity verified that the sorted LKS preparations were 97.1 ± 1.32% pure (*n* = 6).

### Isolation, culture and identification of bone marrow mesenchymal stromal cells

BMMSCs were isolated and cultured using standard protocols [17,23,24]. In brief, erythrocyte-depleted BMCs were plated at a density of 4 × 10^5^ cells/cm^2^ in MesenCult (StemCell Technologies, Vancouver, BC, Canada) supplemented with 100 IU/ml penicillin and 100 μg/ml streptomycin (Invitrogen, Gaithersburg, MD, USA) in a fully humidified atmosphere of 5% CO_2_ in air at 37°C. Culture medium was changed after 24 hours to remove nonadherent cells. Fresh medium was subsequently replaced every 3 days. After 7 days, adherent colony-forming cells were trypsinized, harvested, and immunodepleted of FITC-labeled CD11b^+^, CD14^+^ and CD45^+^ cells using anti-FITC magnetic microbeads (Miltenyi Biotec) according to the manufacturer’s instructions. CD45^–^ cells were replated at a density of 5,000 cells/cm^2^, expanded and passaged weekly for an additional 2 to 3 weeks. Cell purity was assessed by FACS analysis using fluorchrome-labeled antibodies against CD3, CD11b, CD14, CD19, CD31, CD34, CD105, CD106, CD133, CD25, CD44, CD45, CD73, CD80, CD86, CD90, Flk-1, c-Kit, Sca-1, MHC class I and MHC class II (Pharmingen/Becton Dickinson, San Diego, CA USA). BMMSCs at the time of experimentation were >99% CD45^–^ based on FACS analysis.

### Differentiation of bone marrow mesenchymal stromal cells *in vitro*

#### ***Osteogenesis***

Osteoblastic differentiation was induced with slight modification of a previously published protocol [23], by culturing confluent BMMSCs for 3 weeks in complete MesenCult medium (StemCell Technologies) supplemented with 10^–8^ M dexamethasone, 5 mM β-glycerophosphate, and 50 μg/ml ascorbic acid. All osteogenic supplements were obtained from StemCell Technologies. Cultures were incubated at 37°C in a humidified atmosphere of air with 5% CO_2._ Culture medium was exchanged every third day for 3 weeks. Osteogenic differentiation, for secreted calcified extracellular matrix, was detected by Alizarin red staining [23,24].

#### ***Adipogenesis***

Confluent culture BMMSCs were cultured for 3 weeks in complete MesenCult medium (StemCell Technologies) supplemented with 10^–8^ M dexamethasone and 5 μg/ml insulin. All adipogenic supplements were obtained from Sigma-Aldrich (St Louis, MO, USA). Cultures were incubated at 37°C in a humidified atmosphere of air with 5% CO_2._ Culture medium was exchanged every third day for 3 weeks. Adipogenesis was detected by Oil red O staining [23,24].

#### ***Chondrogenesis***

BMMSCs were grown in micromass culture pellets in chondrogenesis induction medium as previously described [25]. Briefly, BMMSCs were seeded as 20 μl drops of (1.6 × 10^5^ cells/drop) onto the center of each well of a six-well culture plate and allowed to attach at 37°C for 2 hours. Subsequently, attached MSC nodules were fed chondrogenic medium containing MesenCult medium (StemCell Technologies) supplemented with 10^–8^ M dexamethasone, 6.25 μg/ml insulin, 50 μg/ml ascorbic acid,1 mM sodium pyruvate, 40 μg/ml proline, 50 mg/ml ITS + Premix (these six reagents purchased from Sigma-Aldrich), and 10 ng/ml transforming growth factor beta-1 (Peprotech, Rocky Hill, NJ, USA). Cultures were incubated at 37°C in a humidified atmosphere of air with 5% CO_2._ Culture medium was exchanged every third day for 3 weeks. Chondrogenic differentiation was detected by Alcian blue staining (Sigma-Aldrich).

### RNA extraction

Total RNA was extracted from freshly isolated bone marrow-derived HSPCs (LKSs) and *in vitro* cultured BMMSCs (passage 2 to 3) as previously described [26]. Briefly, pelleted cells from six independent experimental samples were isolated from pooled BMCs collected from three individual mice. Pelleted cells for each sample were homogenized in Trizol reagent (Invitrogen, Carlsbad, CA, USA) and total RNA was isolated using the standard trizol–chloroform–ethanol extraction procedure. RNA’s were resuspended in 15 μl of 10 mM Tris buffer, pH 7.5. Sample purity, quantity, and quality were assessed by determining the A_260/280_ and A_260/230_ ratios on a Nanodrop Spectrophotometer (NanoDrop Technologies Inc., Wilmington, DE, USA) and by measuring the 28S/18S ribosomal RNA ratio and RNA Integrity Number using an Agilent 2100 BioAnalyzer (Agilent Technologies Inc., Santa Clara, CA, USA). All Agilent RNA integrity values were ≥8.5. Reverse transcription was performed with a Roche 1st Strand Synthesis kit (Roche Diagnostics Corporation, Indianapolis, IN, USA). Briefly, 2.5 μg RNA sample was added to a master mix containing 1× reaction buffer, 5 mM MgCl_2_, 1 mM deoxynucleotide mix, 6.4 μg random primers, 100 units RNase inhibitor, and 40 units AMV reverse transcriptase. Then 10 mM Tris buffer, pH 7.5, was used to reach the 40 μl final reaction volume. The final reaction mixture was then subjected to a single reverse-transcription cycle of 25°C for 10 minutes, 42°C for 60 minutes, 99°C for 5 minutes, and 4°C for at least 10 minutes.

### Real-time quantitative PCR gene profiling for cell signaling mRNA transcripts

qRT-PCR was performed using the ABI Prism 7900HT Sequence Detection System (Applied Biosystems, Foster City, CA, USA). Custom-designed Protein Tyrosine Kinase TaqMan^®^ Low Density Array cards (Applied Biosystems) were used to assess gene expression of key transcripts for RTKs, non-RTKs, cytokine-growth receptors, G-protein coupled receptors, and several other cell signaling molecules. Gene targets were selected based on an extensive review of the literature for well-validated gene expression markers and the availability of Assay of Demand commercial primers (Applied Biosystems). The set of TaqMan^®^ Low Density Array cards was comprised of 96 individual target assays (including respective forward and reverse primers and a dual-labeled probe (5′-6-FAM; 3′-MGB) in quadruplicate on a 384-well card (96 genes per card including two housekeeping genes, 18S and GAPDH). Amplification parameters were as follows: one cycle of 50°C for 2 minutes and 95°C for 10 minutes followed by 40 cycles of 95°C for 30 seconds and 60°C for 1 minute.

### RT-PCR data analysis

RT-PCR data were analyzed using the Sequence Detection System version 2.1 included with the ABI Prism 7900HT SDS and Microsoft Excel. The threshold was manually set and the baseline was set automatically to obtain the threshold cycle (C_t_) value for each target. 18S ribosomal RNA was used as an endogenous housekeeping control gene for normalization. Six independent HSPC and BMBMC experimental samples were run in duplicate wherein C_t_ measurements per samples were normalized using 18S. Relative expression between HSPCs and BMMSCs was determined using the comparative C_t_ method (2^–ΔΔCt^) [27,28]. Results are expressed as the mean ± standard deviation difference in relative expression. Transcription of a particular gene transcript in BMMSCs was considered to be differentially upregulated or downregulated if it was differentially expressed by at least twofold when compared with the expression level in HSPCs, and *vice versa* for the reverse analysis. Assays with C_t_ values greater than 35 cycles were excluded from analysis.

### Validation of qRT-PCR results using FACS analysis for cell surface protein expression

LKSs (HSPCs) and BMMSCs were stained with rat anti-mouse CD45-PE/FITC, Sca-1-PE, c-Kit-FITC, and Flk1-PE (Pharmingen/Becton Dickinson) or rabbit polyclonal anti-human DDR2 (H-108, cross-reacts with mouse), rat anti-mouse PDGFR-α (RM0004-3G28; Santa Cruz Biotechnology, Santa Cruz, CA, USA), primary antibodies followed by PE-labeled goat anti-rabbit and goat anti-rat secondary antibodies, respectively (Pharmingen/Becton Dickinson).

### Statistical analyses

For each mRNA measured in qRT-PCR, replicate C_t_ values for six biological samples were averaged to obtain the mean and standard error of the mean. A paired two-tailed *t* test (analysis of variance) was performed to determine whether the expression was different between the HSPCs and BMBMCs. Individual genes were identified as differentially expressed with ≥2-fold difference between cell types and *P* ≤0.05. Data were analyzed using GraphPad Prism version 4.01 (GraphPad Software, San Diego, CA, USA).

## Results

### Bone marrow-derived HSPC and MSC populations

**Figure 1:**
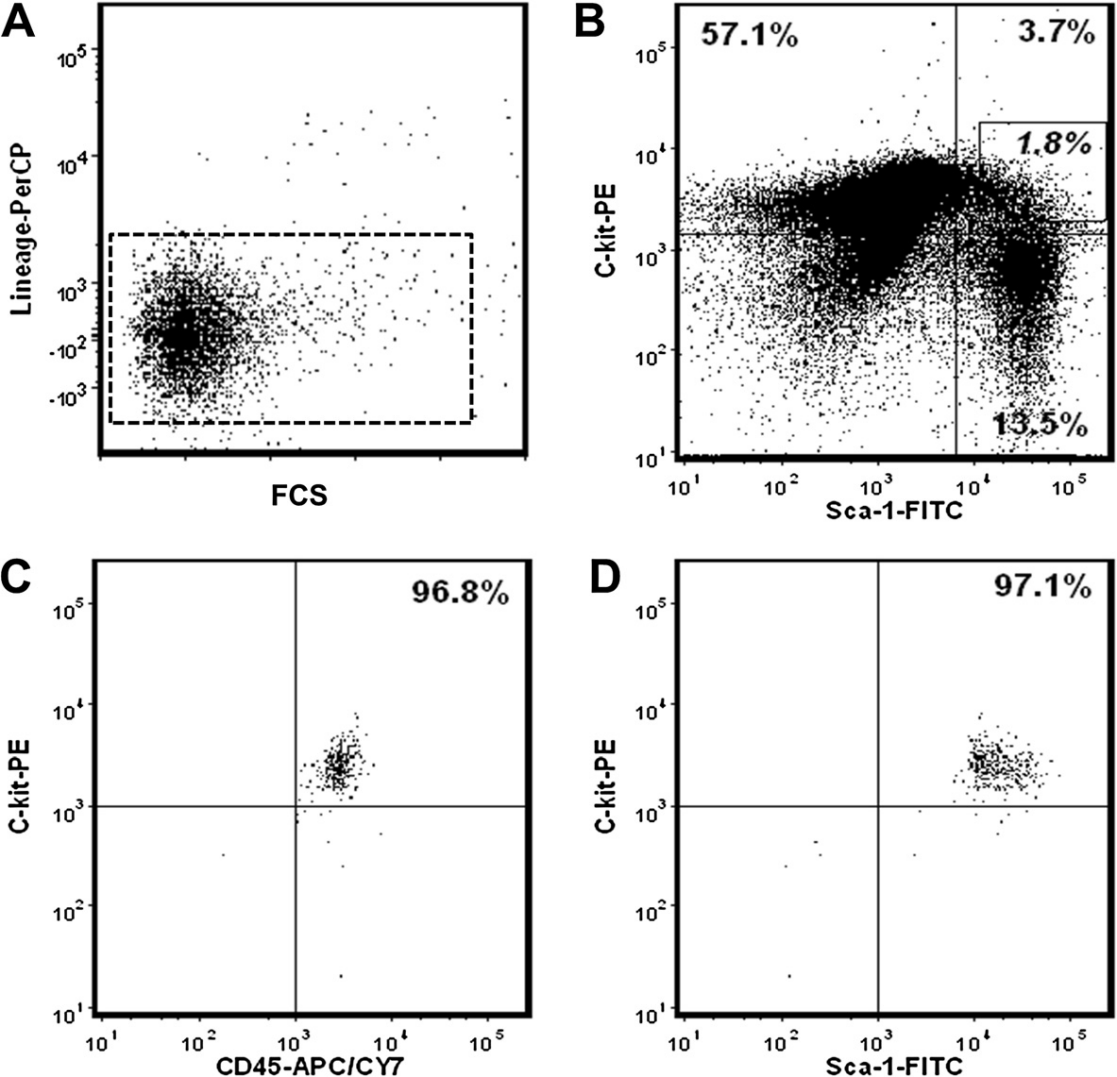
**Enrichment of murine bone marrow hematopoietic stem/progenitor (Lin**^**–**^**c-kit**^**+**^**Sca-1**^**+**^**) cells by fluorescence-activated cell sorting.** Lin^–^ bone marrow cells **(A)** were fluorescence-activated cell sorted for cells expressing high levels of c-kit and Sca-1 **(B)**. Dot plots showing the purity of resorted c-Kit^+^ CD45^+^ cells **(C)** and Sca-1^+^ c-Kit^+^ cells **(D)**. Representative results of six independent HSPC cell sorting preparations are shown. FCS, fetal calf serum; FITC, fluorescein isothiocyanate; PE, phycoerythrin.

To obtain accurate and consistent gene transcription profiles of bone marrow-derived HSPC and MSC populations, we isolated and used highly purified cell populations. Practically all HSPC activity has been shown to be contained within the LKS BMC compartment, which represents 0.05 to 0.1% of total BMCs [29]. LKS cells were isolated by lineage-negative selection (pooled bone marrow from three mice, *n* = 6 separate experiments) followed by double FACS sorting to high purities (98 ± 1.32%; Figure 1A-D). Total RNA from a total of six individual LKS samples was extracted to conduct qRT-PCR gene profiling for cell signaling transcripts in duplicate using a custom-designed Cell Signaling TaqMan^®^ Low Density Array. For each mRNA measured in qRT-PCR, gene expression values were averaged across six biological samples run in technical replicates.

**Figure 2:**
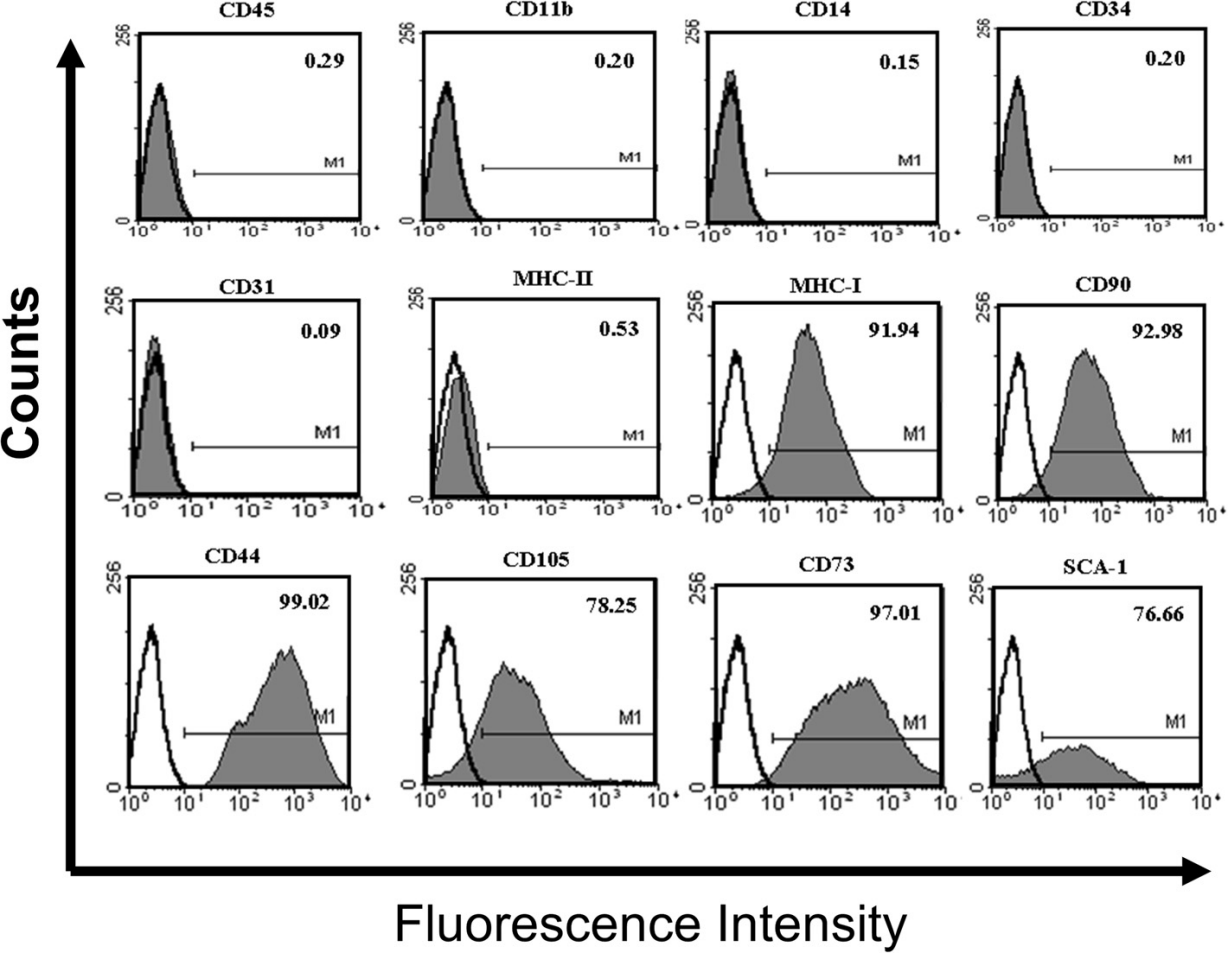
**Immunophenotypic characterization of bone marrow mesenchymal stromal cells by flow cytometry (fluorescence-activated cell sorting).** Fluorescence-activated cell sorting analysis of cell surface markers illustrating that bone marrow-derived mesenchymal stromal cells (BMMSCs) express known stromal–mesenchymal markers such as CD44, CD73, CD90, CD105, and MHC class I and are negative for the hematopoietic cell lineage markers including CD45, CD11b, CD14, CD34, MHC class II and CD31. Unfilled curve, cells stained with isotype control antibody; filled gray curve, staining against each specific cell surface marker.

**Figure 3:**
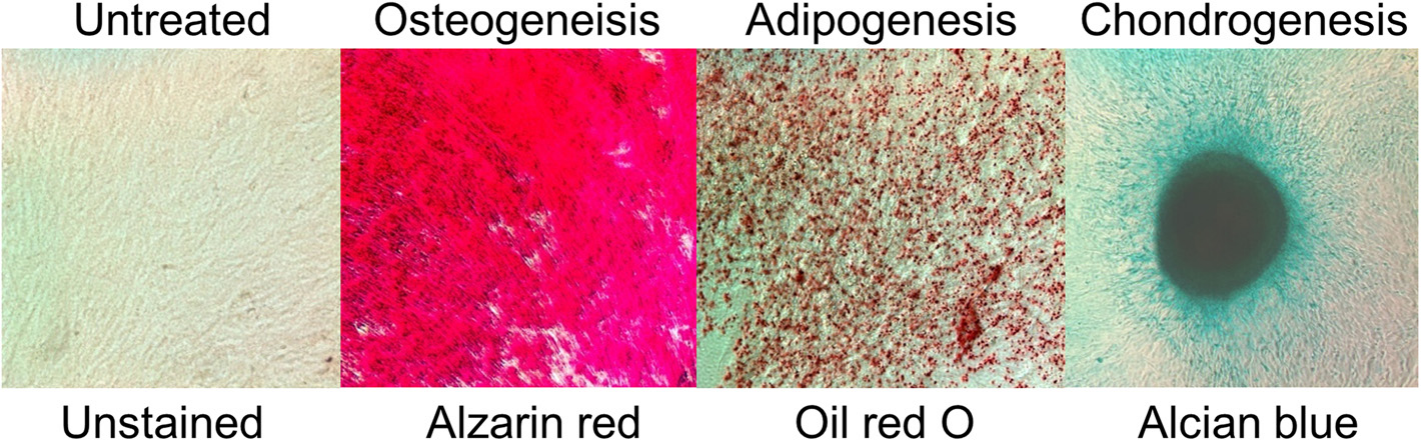
***In vitro *osteogenic, adipogenic and chondrogenic differentiation of bone marrow mesenchymal stromal cells.** Bone marrow-derived mesenchymal stromal cells (BMMSCs; passage 2 to 3) were cultured *in vitro* under osteogenic, adipogenic and chondrogenic induction condition for 3 weeks. Alizarin Red staining shows mineralization deposition, Oil Red O staining demonstrates the generation of lipid-containing adipocytes and Alcian blue staining demonstrates cartilage matrix (magnification 400). Data shown are representative of six independent experiments.

Plastic adherent bone marrow stromal cells were isolated from pooled bone marrow from three mice, propagated for 1 week, hematopoietic cell depleted, and expanded further *in vitro* for 2 to 3 weeks, at which time they reached a stable MSC phenotype by FACS analysis (Figure 2) positive for known stromal–mesenchymal markers such as CD44, CD73, CD90, CD105, and MHC class I and negative for the hematopoietic cell lineage markers including CD45, CD11b, CD14, CD34, MHC class II and CD31. BMMSCs expressed low levels of CD106, Flk-1 and CD133 and were negative for CD3, CD25, CD19, c-Kit (CD117) and for costimulatory molecules CD80 and CD86 (data not shown). To establish that these cells are true MSCs, cells were cultured under various induction conditions to assess their capacity to differentiate into a number of mesodermal lineages. As illustrated in Figure 3, BMMSCs display a multilineage differentiation capacity toward the adipogenic, osteogenic and chondrogenic cell lineages.

### Expression profile of receptor tyrosine kinase genes

To compare gene expression levels within purified HSPC and BMMSC populations, RNA samples from six separate pooled experimental samples were prepared and key transcripts for RTKs, non-RTKs, cytokine-growth receptors, G-protein coupled receptors, and several other cell signaling molecules were assayed in duplicate using qRT-PCR. In rodents, 58 RTKs have been identified. In our study, 37 primer/probe sets for RTK transcripts were included in a customized TaqMan^®^ gene expression array card of total 96 genes (Table 1). Out of these 37 RTK genes, only Aatk (apoptosis associated tyrosine kinase) and Csf1r (colony-stimulating factor 1 receptor) were over expressed, 2.5-fold and 197-fold respectively, in HSPCs in comparison with BMMSCs. In contrast to HSPCs, transcripts for 13 RTK genes (Axl, Mertk, Tyro3, Epha1, Epha2, Epha4, Epha7, Ephb4, Egfr, Fgfr1, Pdgfrb, Met and Ret) were overexpressed 3-fold to 819-fold in BMMSCs. Four RTK transcripts uniquely expressed by HSPCs were Flt3 (FMS-like tyrosine kinase 3), Kit (kit oncogene), Tek (endothelial-specific RTK) and Tie1, whereas Ddr1, Ddr2, Epha3, Ephb3, Fgfr2, Fgfr3, Pdgfra, Mst1r, Ror1 and Ror2 were identified as 10 BMMSC-specific RTK genes. RTK transcripts for Insr (insulin receptor), Flt1 (FMS-like tyrosine kinase 1) and Kdr (kinase insert domain protein receptor) were generally equally expressed in HSPCs and BMMSCs, whereas transcripts for five RTK genes (Epha6, Epha8, Musk, Ros1 and Ntrk2) were not detectable in either HSPCs or BMMSCs.

**Table 1:** Differential gene expression between bone marrow-derived hematopoietic stem/progenitor cells and bone marrow-derived mesenchymal stromal cells

**Gene identification**	**Assay on demand**^**a**^	**∆Ct HSPC**^**b**^	**∆Ct BMMSC**^**b**^	**Fold-change HSPC/BMMSC**^**c**^	***P *** **value**
**RTK gene transcripts**					
Aatk-apoptosis-associated tyrosine kinase	Mm00545697_m1	16.8 ± 0.57	17.91 ± 0.30	2.5	0.009
Axl-AXL receptor tyrosine kinase	Mm00437221_m1	21.03 ± 0.41	11.49 ± 0.32	-818.7	0.0001
Mertk-c-mer proto-oncogene tyrosine kinase	Mm00434920_m1	23.04 ± 0.76	18.68 ± 0.41	-21.7	0.0001
Tyro3-TYRO3 protein tyrosine kinase 3	Mm00444547_m1	21.49 ± 0.62	15.83 ± 0.38	-52	0.0001
Ddr1-discoidin domain receptor family, member 1	Mm00432251_m1	ND	21.20 ± 0.49		
Ddr2-discoidin domain receptor family, member 2	Mm00445615_m1	ND	11.46 ± 1.94		
Egfr-epidermal growth factor receptor	Mm00433023_m1	23.44 ± 0.64	16.89 ± 0.48	-88.8	0.0001
Epha1-Eph receptor A1	Mm00445804_m1	23.88 ± 0.83	20.73 ± 0.42	-9.2	0.0001
Epha2-Eph receptor A2	Mm00438726_m1	19.20 ± 0.96	17.34 ± 0.46	-3.8	0.0001
Epha3-Eph receptor A3	Mm00580743_m1	ND	22.18 ± 0.33		
Epha4-Eph receptor A4	Mm00433056_m1	22.17 ± 0.49	20.76 ± 0.41	-2.8	0.0001
Epha6-Eph receptor A6	Mm00433094_m1	ND	ND		
Epha7-Eph receptor A7	Mm00833876_m1	22.06 ± 1.10	17.57 ± 0.29	-22.9	0.0001
Epha8-Eph receptor A8	Mm00433106_m1	ND	ND		
Ephb3-Eph receptor B3	Mm00802553_m1	ND	16.69 ± 0.79		
Ephb4-Eph receptor B4	Mm00438750_m1	22.21 ± 0.46	15.50 ± 0.33	-107.3	0.0001
Fgfr1-fibroblast growth factor receptor 1	Mm00438923_m1	20.91 ± 1.27	15.95 ± 0.20	-36.5	0.0001
Fgfr2-fibroblast growth factor receptor 2	Mm00438941_m1	ND	20.55 ± 0.40		
Fgfr3-fibroblast growth factor receptor 3	Mm00433294_m1	ND	20.32 ± 0.48		
Insr-insulin receptor	Mm00439693_m1	15.97 ± 0.43	15.82 ± 0.29	-1.1	0.3273
Met-met proto-oncogene	Mm00434924_m1	18.25 ± 0.38	14.36 ± 0.49	-15.9	0.0001
Mst1r-macrophage stimulating 1 receptor (c-met-related tyrosine kinase)	Mm00436365_m1	ND	24.49 ± 1.08		
Musk-muscle, skeletal, receptor tyrosine kinase	Mm00448006_m1	ND	ND		
Csf1r-colony stimulating factor 1 receptor	Mm00432689_m1	14.89 ± 0.43	22.45 ± 0.43	197.3	0.0001
Flt3-FMS-like tyrosine kinase 3	Mm00438996_m1	17.68 ± 0.87	ND		
Kit-kit oncogene	Mm00445212_m1	15.21 ± 0.63	ND		
Pdgfra-platelet derived growth factor receptor, alpha polypeptide	Mm00440701_m1	ND	16.71 ± 0.66		
Pdgfrb-platelet derived growth factor receptor, beta polypeptide	Mm00435546_m1	19.98 ± 0.60	13.83 ± 0.24	-72.4	0.0001
Ret-ret proto-oncogene	Mm00436304_m1	22.27 ± 0.64	20.56 ± 0.41	-3.4	0.0001
Ror1-receptor tyrosine kinase-like orphan receptor 1	Mm00443462_m1	ND	18.75 ± 0.37		
Ror2-receptor tyrosine kinase-like orphan receptor 2	Mm00443470_m1	ND	19.22 ± 0.30		
Ros1-Ros1 proto-oncogene	Mm00803362_m1	ND	ND		
Tek-endothelial-specific receptor tyrosine kinase	Mm00443242_m1	18.52 ± 1.21	ND		
Tie1-tyrosine kinase receptor 1	Mm00441786_m1	20.39 ± 0.84	ND		
Ntrk2-neurotrophic tyrosine kinase, receptor, type 2	Mm00435422_m1	ND	ND		
Flt1-FMS-like tyrosine kinase 1	Mm00438980_m1	21.13 ± 0.41	20.52 ± 0.49	-1.6	0.0032
Kdr-kinase insert domain protein receptor	Mm00440099_m1	23.48 ± 0.60	23.31 ± 0.70	-1.3	0.5296
**Non-RTK transcripts**					
Abl1-v-abl Abelson murine leukemia oncogene 1	Mm00802038_g1	16.54 ± 0.54	14.93 ± 0.14	-3	0.0001
Tnk1-tyrosine kinase, non-receptor, 1	Mm00840782_g1	ND	ND		
Tnk2-tyrosine kinase, non-receptor, 2	Mm00450301_m1	17.25 ± 0.34	15.88 ± 0.25	-2.6	0.0001
Matk-megakaryocyte-associated tyrosine kinase	Mm00440268_m1	17.49 ± 0.83	22.68 ± 0.58	41.9	0.0001
Ptk2-PTK2 protein tyrosine kinase 2	Mm00433209_m1	20.21 ± 0.90	14.67 ± 0.28	-47.1	0.0001
Fert2-fer (fms/fps related) protein kinase, testis specific 2	Mm00484303_m1	17.74 ± 0.48	15.11 ± 0.22	-6.3	0.0001
Fes-feline sarcoma oncogene	Mm00802572_g1	13.51 ± 0.47	17.67 ± 0.45	18.8	0.0001
Frk-fyn-related kinase	Mm00456656_m1	ND	18.56 ± 0.54		
Srmssrc-related kinase lacking C-terminal regulatory tyrosine	Mm00441546_m1	ND	ND		
Jak1-Janus kinase 1	Mm00600614_m1	13.23 ± 0.54	12.47 ± 0.43	-1.8	0.0009
Jak2-Janus kinase 2	Mm00434561_m1	14.69 ± 0.63	15.81 ± 0.25	2.4	0.0001
Jak3-Janus kinase 3	Mm00439962_m1	16.78 ± 0.62	17.93 ± 0.20	2.4	0.0001
Fgr-Gardner-Rasheed feline sarcoma viral (Fgr) oncogene homolog	Mm00438949_m1	14.67 ± 1.61	ND		
Fyn-Fyn proto-oncogene	Mm00433373_m1	17.01 ± 0.54	14.57 ± 0.40	-5.6	0.0001
Hck-hemopoietic cell kinase	Mm00439302_m1	14.2 ± 0.76	22.11 ± 0.54	275.1	0.0001
Lck-lymphocyte protein tyrosine kinase	Mm00802897_m1	19.42 ± 0.49	23.04 ± 0.43	13	0.0001
Lyn-Yamaguchi sarcoma viral (v-yes-1) oncogene homolog	Mm00802933_m1	13.28 ± 0.87	19.56 ± 0.51	93.1	0.0001
Bmx-BMX non-receptor tyrosine kinase	Mm00515368_m1	14.73 ± 1.07	ND		
Btk-Bruton agammaglobulinemia tyrosine kinase	Mm00442712_m1	15.05 ± 1.20	24.92 ± 0.76	972.4	0.0001
Tec-cytoplasmic tyrosine kinase, Dscr28C related (Drosophila)	Mm00443230_m1	16.2 ± 0.34	18.24 ± 0.33	4.2	0.0001
Txk-TXK tyrosine kinase	Mm00443280_m1	20.98 ± 0.50	ND		
Syk-spleen tyrosine kinase	Mm00441649_m1	12.74 ± 0.57	17.10 ± 0.21	22.1	0.0001
Zap70-zeta-chain (TCR) associated protein kinase	Mm00494255_m1	19.49 ± 1.30	ND		
**G-protein coupled receptor transcripts**					
Agtrl1-angiotensin receptor-like 1	Mm00442191_s1	19.96 ± 0.56	20.22 ± 0.88	1.3	0.3972
Ednrb-endothelin receptor type B	Mm00432989_m1	22.96 ± 1.06	22.70 ± 0.67	-1	0.4802
Ccr1-chemokine (C-C motif) receptor 1	Mm00438260_s1	13.33 ± 0.63	19.78 ± 1.13	95.6	0.0001
Ccr3-chemokine (C-C motif) receptor 3	Mm00515543_s1	19.93 ± 0.36	20.03 ± 1.10	1.1	0.7675
Ccr4-chemokine (C-C motif) receptor 4	Mm00438271_m1	23.9 ± 1.24	ND		
Ccr7-chemokine (C-C motif) receptor 7	Mm00432608_m1	18.32 ± 1.50	ND		
Ccr8-chemokine (C-C motif) receptor 8	Mm00843415_s1	21.55 ± 0.44	19.71 ± 0.83	-4.1	0.0001
Cxcr3-chemokine (C-X-C motif) receptor 3	Mm00438259_m1	20.74 ± 0.36	ND		
Blr1 (Cxcr5)-Burkitt lymphoma receptor 1	Mm00432086_m1	20.88 ± 1.00	ND		
Cmkor1 (Cxcr7)-chemokine orphan receptor 1	Mm00432610_m1	23.68 ± 1.01	15.52 ± 0.35	-293.2	0.0001
**Cytokine receptor transcripts**					
Acvr1-activin A receptor, type 1	Mm00431645_m1	17.98 ± 0.83	13.57 ± 0.45	-22.2	0.0001
Acvrl1-activin A receptor, type II-like 1	Mm00437432_m1	20.73 ± 0.60	19.32 ± 0.67	-2.9	0.0001
Bmpr1a-bone morphogenetic protein receptor, type 1A	Mm00477650_m1	17.28 ± 0.95	14.75 ± 0.22	-5.9	0.0001
Il3ra-interleukin 3 receptor, alpha chain	Mm00434273_m1	18.52 ± 0.53	18.40 ± 0.44	-1.1	0.5524
Il6ra-interleukin 6 receptor, alpha	Mm00439653_m1	14.54 ± 0.38	16.40 ± 0.51	3.8	0.0001
Il7r-interleukin 7 receptor	Mm00434295_m1	17.11 ± 0.24	24.38 ± 0.75	155.9	0.0001
Csf2ra-colony stimulating factor 2 receptor, alpha	Mm00438331_g1	14.46 ± 0.40	19.04 ± 0.22	24.9	0.0001
Csf2rb2-colony stimulating factor 2 receptor, beta 2	Mm00655763_m1	17.33 ± 0.38	ND		
Csf3r-colony stimulating factor 3 receptor	Mm00432735_m1	11.85 ± 1.43	ND		
Epor-erythropoietin receptor	Mm00833882_m1	19.42 ± 1.07	22.59 ± 0.85	11.6	0.0001
Lifr-leukemia inhibitory factor receptor	Mm00442940_m1	17.89 ± 0.64	16.76 ± 0.36	-2.3	0.0001
Mpl-myeloproliferative leukemia virus oncogene	Mm00440310_m1	14.86 ± 1.69	ND		
Ngfr-nerve growth factor receptor (TNFR superfamily, member 16)	Mm00446294_m1	ND	11.96 ± 0.30		
Osmr-oncostatin M receptor	Mm00495424_m1	ND	15.97 ± 0.24		
Tnfrsf1a-tumor necrosis factor receptor superfamily, member 1a	Mm00441875_m1	13.49 ± 0.36	13.91 ± 0.24	1.4	0.0023
**Other signaling molecule transcripts**					
Abcb1b-ATP-binding cassette, sub-family B (MDR/TAP), member1B	Mm00440736_m1	18.64 ± 0.64	17.73 ± 0.38	-1.9	0.0003
Mrc1-mannose receptor, C type 1	Mm00485148_m1	20.76 ± 0.47	22.00 ± 0.62	2.5	0.0001
Gata4-GATA binding protein 4	Mm00484689_m1	ND	20.10 ± 0.31		
Hoxb4-homeo box B4	Mm00657964_m1	17.88 ± 0.50	17.28 ± 0.59	-1.7	0.0134
Ptprc-protein tyrosine phosphatase, receptor type, C	Mm00448463_m1	ND	ND		
Gas6-growth arrest specific 6	Mm00490378_m1	23.45 ± 1.02	14.90 ± 0.26	-381.1	0.0001
Lgals9-lectin, galactose binding, soluble 9	Mm00495295_m1	13.67 ± 0.82	17.69 ± 0.25	18.9	0.0001
Sca1-spinocerebellar ataxia 1 homolog (human)	Mm00485928_m1	17.08 ± 0.23	15.71 ± 0.29	-2.6	0.0001
Spp1-secreted phosphoprotein 1	Mm00436767_m1	17.91 ± 0.33	9.23 ± 0.44	-424.9	0.0001

Gene differential expression was considered significant with *P* <0.05. Mean ± standard deviation of six independent HSPC and BMMSC preparations are shown. BMMSC, bone marrow-derived mesenchymal stromal cell; C_t_, cycle threshold; HPSC, hematopoietic stem/progenitor cell; ND, not detectable; RTK, receptor tyrosine kinase. ^a^Quantitative PCR was performed on an ABI PRISM 7900HT Sequence Detection System using a custom-made TaqMan^®^ Low Density Array. mRNA transcripts were evaluated with TaqMan^®^ Probes commercially available as Assay on Demand (Applied Biosystems, Foster City, CA, USA) with optimized primer and probe concentrations.

^b^Mean ± standard deviation mRNA level of each gene in each cell population (six individual cell samples run in duplicate) were first normalized to the expression of 18S RNA in that sample.

^c^Mean fold-changes in gene transcript expression levels between HSPCs and BMMSCs were evaluated with 2^–ΔΔCt^.

### Expression profile of cytoplasmic non-tyrosine kinase genes

Out of 32 murine non-RTK genes, we included 23 genes in our present study (Table 1). Among the non-RTK genes evaluated, 10 genes (Btk, Tec, Hck, Lck, Lyn, Jak2, Jak3, Matk, Fes, and Syk) were upregulated by twofold to 972-fold in HSPCs in comparison with BMMSCs and five non-RTK genes (Abl1, Fert2, Fyn, Ptk2, and Tnk2) were overexpressed threefold to 47-fold in BMMSCs. Moreover, four non-RTK genes (Bmx, Txk, Fgr and Zap70) were found to be exclusively expressed in HSPCs. Fyn was only detectable in BMMSCs, while Jak1was similarly expressed in HSPCs and BMMSCs. Transcripts for the non-RTK genes Tnk1 and Srms were not detectable in either HSPCs or BMMSCs.

### Expression profile of G-protein coupled receptor genes

G-protein coupled receptors comprise a large protein family of transmembrane receptors that transduce extracellular stimuli into intracellular signals through their interaction with heterotrimeric G proteins [30]. All 19 distinct mammalian chemokine receptors are the members of the large protein family G-protein coupled receptors [31]. We analyzed 10 G-protein coupled receptor transcripts, including eight chemokine receptors (Ccr1, Ccr3, Ccr4, Ccr7, Ccr8, Cxcr3, Cxcr5 and Cxcr7), in our custom-designed gene expression array profile (Table 1). Ccr1 was expressed 96-fold more in HSPCs than in BMMSCs, and gene transcripts for Ccr8 and Cmkor1 (Cxcr7) were upregulated fourfold and 293-fold respectively in BMMSCs when compared with HSPCs. Expression of Ccr4, Ccr7, Cxcr3 and Blr1 (Cxcr5) were limited to HSPCs, while transcript expression for Ccr3, Agtrl1 (angiotensin receptor-like 1) and Ednrb (endothelin receptor type B) were similarly expressed in both HSPCs and BMMSCs.

### Expression profile of cytokine receptor genes

Cytokine receptors are transmembrane receptors expressed on the surface of a wide range of cells that recognize and respond to cytokines; however, cytokine receptors lack intrinsic protein tyrosine activity found in many other receptors [32]. Signaling through cytokine receptors depends upon their interaction with Janus kinases, which couple ligand binding to tyrosine phosphorylation of signaling recruited to the receptor complex [33]. Fifteen members of the type-1 cytokine receptor family (CRF1) mostly comprising the hematopoietin cytokine receptors (Il3ra, Il6ra, Il7r, Csf2ra, Csf2rb, Csf3r, Epor, Osmr, Lifr, Mpl, Ngfr, Tnfrsf1a, Acvr1, Acvrl1 and Bmpr1a) were included in our differential gene expression assessment. Out of these 15 cytokine receptor genes, Il6ra, Il7r, Csf2ra and Epor were upregulated in HSPCs by fourfold, 156-fold, 25-fold and 12-fold respectively when compared with transcript expression levels in BMMSCs (Table 1). Acvr1, Acvrl1, Bmpr1a and Lifr were overexpressed by 22-fold, 3-fold, 6-fold and 2-fold more in MSCs. Csf2rb, Csf3r and Mpl were exclusively expressed in HSPCs. Ngfr and Osmr expression was restricted to MSCs, and IL3ra and Tnfrsf1a were similarly expressed in both HSPCs and BMMSCs.

### Expression profile of transcripts for other cell signaling molecules

A few other cell signaling targets genes were evaluated and were differentially expressed in HSPC and BMMSC populations (Table 1). Transcript expression of the leukocyte common antigen (PTPRC/CD45) was restricted to HSPCs. Genes transcripts for Mrc1 (mannose receptor, C type 1) and Lgals9 (lectin, galactose binding, soluble 9) were overexpressed in HSPCs by 2.5-fold and 19-fold respectively in comparison with the transcript levels in BMMSCs (Table 1), while Gas6 (growth arrest specific 6), Sca1 (spinocerebellar ataxia 1), and Spp1 (secreted phosphoprotein-1) expression levels were significantly higher, 381-fold, 3-fold, and 425-fold respectively, in BMMSCs. Furthermore, Gata4 (GATA binding protein 4), a transcription factor, was exclusively expressed in BMMSCs, whereas the expression levels of the transcription factor Hoxb4 (homeobox B4) and Abcb1b (ATP-binding cassette, sub-family B (MDR/TAP), member 1B) were similar in both HSPCs and BMMSCs.

### Flow cytometry validation of RT-PCR array data

**Figure 4:**
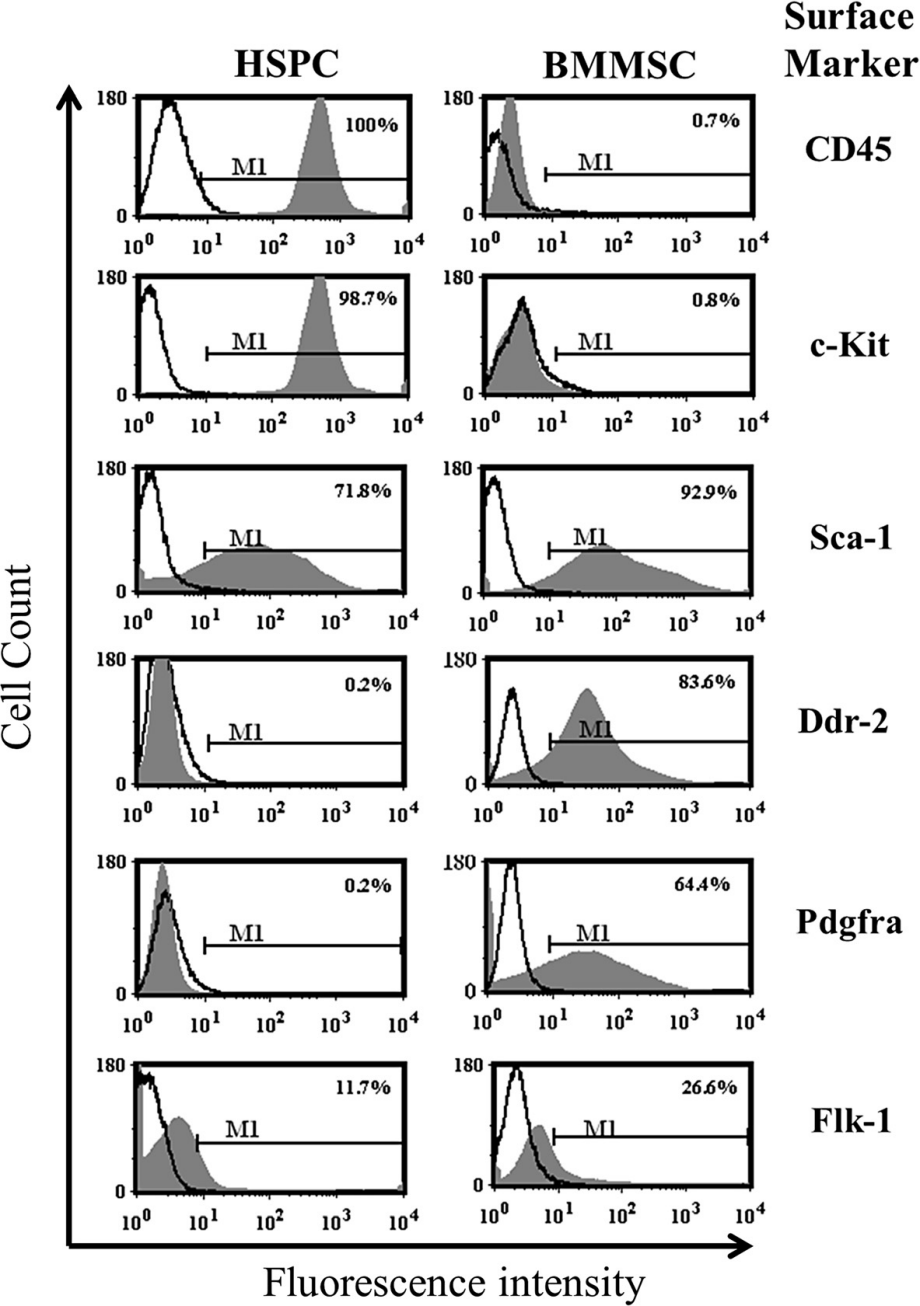
**Cell surface protein expression on undifferentiated hematopoietic stem/progenitor cells and bone marrow-derived mesenchymal stromal cells.** Cell surface expression of CD45, cKit, Sca-1, Kdr (Flk1/Vegfr2), Ddr2 and Pdgfra on undifferentiated Lin^–^c-Kit^+^Sca-1^+^ cells (hematopoietic stem/progenitor cells (HSPCs)) and bone marrow-derived mesenchymal stromal cell (BMMSCs; passage 2) via flow cytometric analysis. Unfilled curve, cells stained with isotype control antibody; filled gray curve, staining against each specific protein.

To validate the results obtained by qRT-PCR microarray analysis, we selected six target genes that are differentially expressed, and assessed their comparative expression corresponding cell surface protein levels using flow cytometric analysis (Figure 4). Our mRNA transcript results demonstrate the receptors for collagen (Ddr2) and platelet-derived growth factor (Pdgfra) were exclusively expressed only on BMMSCs that lack c-kit and CD45 expression (Figure 2), whereas both populations of cells expressed transcripts and cell surface protein for Sca-1 and KDR (Flk-1/Vegfr2).

## Discussion

Bone marrow HSPCs and BMMSCs share a common microenvironmental niche wherein intercellular and intracellular network signaling communications direct stem cell fate activation, proliferation, development, and tissue differentiation [34,35]. Limited comparative information is available on the molecular signaling behavior of undifferentiated BMMSCs and HSPCs. Defining the signaling mechanisms expressed in adult undifferentiated stem cells is an essential step toward understanding the developmental and regenerative capabilities. Here we report a comprehensive evaluation, of mRNA gene transcripts for 94 signaling molecules, in which 11 transcripts were equally expressed in both HSPCs and BMMSCs, 19 overexpressed in HSPCs compared with BMMSCs, 27 overexpressed BMMSCs compared with HSPCs, 16 expressed only in HSPCs, 14 expressed only in BMMSCs and seven expressed in neither cell population. To our knowledge, this is the first study to report simultaneous determination of multiple cell signaling molecules in highly purified undifferentiated stem cell populations under standardized conditions. Flow cytometric analysis showed that the transcriptional levels of CD45, c-kit, Sca-1, KDR (Flk-1/Vegfr2), Pdgfra, and Ddr2 were consistent with the cell surface translational levels of protein expression.

Of the 90 PTKs, 58 are categorized as RTKs and 32 as cytoplasmic non-RTKs [14]. Of the 37 RTK gene transcripts we evaluated, 23 gene transcripts were either exclusively confined to or more highly expressed in BMMSCs. Transcripts for Aatk, and Csfr1were more highly expressed in HSPCs than in BMMSCs, while transcripts Flt3, Kit, Tek, and Tie1were found to be exclusively expressed in HSPCs, all known receptors for ligands that have been shown to be important in primitive HSPC survival, quiescence, activation, proliferation, mobilization and/or differentiation [36-41]. In contrast, we found in BMMSCs a different set of transcripts for genes encoding signaling receptors linked to stem cell survival and growth (Axl, Pdgfr and Egfr), self-renewal (Egfr and Ephr), maintenance of stem cells in the dedifferentiate state (Egfr, Fgfr), and recruitment of cells to injured tissue (Met, Mstl1R, and Pdgfr) [42-47]. Furthermore signaling molecules that modulate osteogenesis/chondrogenesis (Ror1, Ror2, Ddr1, and Ddr2) and neuronal cell development (Ret) were either exclusively or differentially expressed in BMMSCs. We show transcription of Gas6, a secreted vitamin-K-dependent protein ligand for Axl, Mertk, and Tyro3 known to play a role in reversible cell growth arrest, survival, proliferation, cell adhesion, long-term hematopoiesis, and erythropoiesis [48-50], is ubiquitously expressed in HSPCs and BMMSCs, although transcript expression was 381-fold greater in BMMSCs.

Non-RTKs are integral components of the signaling cascades triggered by RTKs and by other cell surface receptors such as G protein-coupled receptors and growth factor/cytokine receptors of the immune system [51]. Not surprisingly, transcripts for Csf1r, Csf2ra, Csf2rb2, Csf3r, IL6ra, IL7r, Epor, Mpl, flt3, Kit, Tie-1 and Tek receptors for the corresponding cytokine ligands M-CSF, GM-CSF, G-CSF, IL-6, IL-7, EPO, TPO, FLT3L, SCF, and angiopoietin-1 were found to be HSPC specific, whereas receptors of leukemia inhibitor factor (Lifr), nerve growth factor (Ngfr) and oncostatin-M (Osmr, stimulates BMMSCs to produce stromal-derived growth factor) were mainly expressed in BMMSCs. As hematopoietic supportive cells, BMMSCs constitutively expressed transcripts for M-CSF, IL-6, IL-11, LIF, SCF and Flt3 ligand, and inflammatory cytokine stimulation of BMMSCs with IL-1α induces G-CSF, and GM-CSF expression [52]. These findings highlight the importance of BMMSCs in the context of the HSPC niche where they support HSPC survival (anti-apoptotic action) and quiescence [53]. Furthermore, we found that the expression of G-protein chemokine receptors for the cell trafficking molecules MIP-1, RANTES, TARC, and MCP-1 (Cccr4), MIP-3β (Ccr7) and IP-10, I-TAC and Mig (Cxcr3) was exclusive in HSPCs. These data suggest and are consistent with the notion that quiescent HSPCs are poised for mobilization.

Consistent with the RTK and non-RTK findings, HSPCs were notably enriched in Tec kinases (Tec, Btk, Bmx, and Txk), SRC kinases (Fgr and Lck), SFK kinases (Hck), Syk Kinases (Syk and Zap-70), Janus kinase/STAT kinases (Jak2 and Jak3) and c-fes kinases (Fes). These intracellular regulated transcripts are known to be important in early HSPC decisions, and may play a key role in HSPC self-renewal, quiescence and lineage-specific differentiation. In contrast, BMMSCs expressed higher transcript levels of Abl1, Fert2, Fyn Ptk2, Tnk2, and Frk, which have been shown in other cell types to have cytoplasmic and/or nuclear regulatory functions in during cell differentiation, cell remodeling, cell division, cell adhesion and cell migration [54-59]; however, their roles in BMMSCs are unknown and further evaluation is needed.

Our findings in this report are subject to several limitations. First, we compared cell signaling receptors of cultured early passaged BMMSCs to freshly isolated HSPCs. It is possible that some of the differential expression in these genes is solely due to the fact that BMMSCs were cultured whereas the HSPCs were not. This may account for an over-representation of RTKs in BMMSCs compared with HSPCs. Second, it is accepted that *in vivo* conditions are different from the *in vitro* experimental culture conditions wherein most of the niche microenvironmental conditions are absent. Third, gene expression is under regulatory control at many different stages, and therefore it is difficult to equate mRNA levels with gene expression levels. Fourth, future studies are needed to determine the signaling profiles during times of stress, injury, inflammation or repair. Lastly, the gene expression data generated and the conclusions need to be verified *in situ* in localized cells at specific anatomic sites using immunochemistry and laser capture microdissection or other techniques [60].

## Conclusion

In this study, we conducted a comparative analysis of gene transcripts for a number of cell signaling receptors in highly purified undifferentiated HSPCs and BMMSCs. Clearly the expression of a number of these genes overlaps between HSPCs and BMMSCs, but comparative analysis of the gene profiles showed that there are a substantial number of gene transcripts that are distinct or more highly expressed in specific stem cell populations. Evaluating and characterizing the role of these genes in regulating stem behavior in terms of cell quiescence, proliferative capacity, mobility and differentiation potential will be critical to better understanding the developmental and regenerative capabilities of HSPCs and BMMSCs and their potential application in cell-based therapies. A network analysis of RTKs differentially expressed by BMMSCs and of non-RTKs differentially expressed by HSPCs could yield insights into the mechanisms for phosphoprotein networks used by these cells. This information could be potentially valuable for designing media for the efficient expansion of these cells or understanding mechanisms that BMMSCs use to regulate HSPC growth and survival.

## Abbreviations

BMC: Bone marrow cell; BMMSC: Bone marrow-derived mesenchymal stromal cell; Ct: Threshold cycle; FACS: Fluorescence-activated cell sorting; FITC: Fluorescein isothiocyanate; HSPC: Hematopoietic stem/progenitor cell; LKS: Lineage-negative, c-Kit-positive, Sca-1-positive cell; mAb: Monoclonal antibody; MSC: Mesenchymal stromal cell; PE: Phycoerythrin; PTK: Protein tyrosine kinase; qRT-PCR: Quantitative real-time polymerase chain reaction; RTK: Receptor tyrosine kinase; RT-PCR: Reverse transcription polymerase chain reaction.

## Competing interests

The authors declare that they have no competing interests.

## Authors’ contributions

KA designed and performed the experiments, collected and analyzed the data, and assisted in the writing of the manuscript. TAD conceived the study, designed the custom low-density array targets and gene target selections, and supervised the study, including experiment design, data analysis and writing of the manuscript. Both authors read and approved the final manuscript.

## Authors’ information

The authors are employees of the US Government. This work was prepared as part of their official duties. Title 17 U.S.C. *§*105 provides that ‘Copyright protection under this title is not available for any work of the United States Government.’ Title 17 U.S.C *§*101 defined a US Government work as a work prepared by a military service member or employees of the US Government as part of that person’s official duties. The opinions or assertions contained in this paper are the private views of the authors and are not to be construed as reflecting the views, policy or positions of the Department of the Navy or Army, Department of Defense nor the US Government. The experiments reported herein were conducted in compliance with the Animal welfare Act and in accordance with the principles set forth in the current edition of the *Guide for Care and Use of Laboratory Animals*, Institute for Laboratory Animal Resources, National Research Council, National Academy Press, 1996.
